# The Molecular Neurobiology of Twelve Steps Program & Fellowship: Connecting the Dots for Recovery

**DOI:** 10.17756/jrds.2015-008

**Published:** 2015-03-13

**Authors:** Kenneth Blum, Benjamin Thompson, Zsolt Demotrovics, John Femino, John Giordano, Marlene Oscar-Berman, Scott Teitelbaum, David E. Smith, A. Kennison Roy, Gozde Agan, James Fratantonio, Rajendra D. Badgaiyan, Mark S. Gold

**Affiliations:** 1 Department of Psychiatry, School of Medicine and McKnight Brain Institute, University of Florida, Gainesville, FL, USA; 2 Department of Addiction Research and Therapy, Malibu Beach Recovery Center, Malibu Beach, CA, USA; 3 Dominion Diagnostics, Inc., North Kingstown, RI, USA; 4 IGENE, LLC., Austin, TX, USA; 5 RDSolutions, Del Mar, CA, USA; 6 Behavioral Neuroscience Program, Boston University School of Medicine, and Boston VA Healthcare System, Boston, MA, USA; 7 Eötvös Loránd University, Institute of Psychology, Budapest, Hungary; 8 Meadows Edge Recovery Center, North Kingstown, RI, USA; 9 National Institute for Holistic Medicine, North Miami Beach, FL, USA; 10 Departments of Psychiatry, Neurology, and Anatomy & Neurobiology, Boston University School of Medicine, and Boston VA Healthcare System, Boston, MA, USA; 11 Institute of Health & Aging, University of California at San Francisco, San Francisco, CA, USA; 12 Biobehavioral Medical Corporation, Metairie, LA, USA; 13 Department of Psychiatry, University of Minnesota College of Medicine, Minneapolis, MN, USA; 14 Director of Research, Drug Enforcement Administration (DEA) Educational Foundation, Washington, D.C, USA; 15 Departments of Psychiatry & Behavioral Sciences at the Keck, University of Southern California, School of Medicine, CA, USA

**Keywords:** 12 steps, Fellowship, Spirituality, Dopamine agonistic modalities (DAM), God, Genetic testing, Neuroepigenetics, Reward deficiency syndrome (RDS)

## Abstract

There are some who suggest that alcoholism and drug abuse are not diseases at all and that they are not consequences of a brain disorder as espoused recently by the American Society of Addiction Medicine (ASAM). Some would argue that addicts can quit on their own and moderate their alcohol and drug intake. When they present to a treatment program or enter the 12 Step Program & Fellowship, many addicts finally achieve complete abstinence. However, when controlled drinking fails, there may be successful alternatives that fit particular groups of individuals. In this expert opinion, we attempt to identify personal differences in recovery, by clarifying the molecular neurobiological basis of each step of the 12 Step Program. We explore the impact that the molecular neurobiological basis of the 12 steps can have on Reward Deficiency Syndrome (RDS) despite addiction risk gene polymorphisms. This exploration has already been accomplished in part by Blum and others in a 2013 Springer Neuroscience Brief. The purpose of this expert opinion is to briefly, outline the molecular neurobiological and genetic links, especially as they relate to the role of epigenetic changes that are possible in individuals who regularly attend AA meetings. It begs the question as to whether “12 steps programs and fellowship” does induce neuroplasticity and continued dopamine D2 receptor proliferation despite carrying hypodopaminergic type polymorphisms such as DRD2 A1 allele. “Like-minded” doctors of ASAM are cognizant that patients in treatment without the “*psycho-social-spiritual trio*,” may not be obtaining the important benefits afforded by adopting 12-step doctrines. Are we better off with coupling medical assisted treatment (MAT) that favors combining dopamine agonist modalities (DAM) as possible histone-deacetylase activators with the 12 steps followed by a program that embraces either one or the other? While there are many unanswered questions, at least we have reached a time when “science meets recovery,” and in doing so, can further redeem joy in recovery.

## Introduction

The molecular neurobiological aspects of The Twelve Step Program adopted by self-help groups such as Alcoholics Anonymous (AA) and Narcotics Anonymous (NA) are the focus of this expert opinion. The purpose is to inform the addiction community that based on newly discovered functions of the reward circuitry of the brain, the neurobiological mechanisms at work within the 12-step doctrines may be understood.

We are cognizant that the one hundred alcoholics who developed these steps in the early to late 1930’s did so, empirically, without the tools of science we have today. The brain was a real mystery, very little was known about its workings especially the role of neurotransmitters and reward circuitry. Through the advent of 21^st^ century science and medicine, especially neuroimaging technologies, science has finally caught up with “The 12 Step Program and Fellowship”. The mysteries that link the functioning of the brain and reward are being unraveled.

Understanding of the neuro-molecular biological underpinnings of The 12 steps and the work of various groups such as Al-Anon may indeed be a new and important step on the road to becoming and remaining clean and sober. Embracing principles of molecular neurobiology could ultimately lead to a better quality of life in recovery.

**Figure 1:**
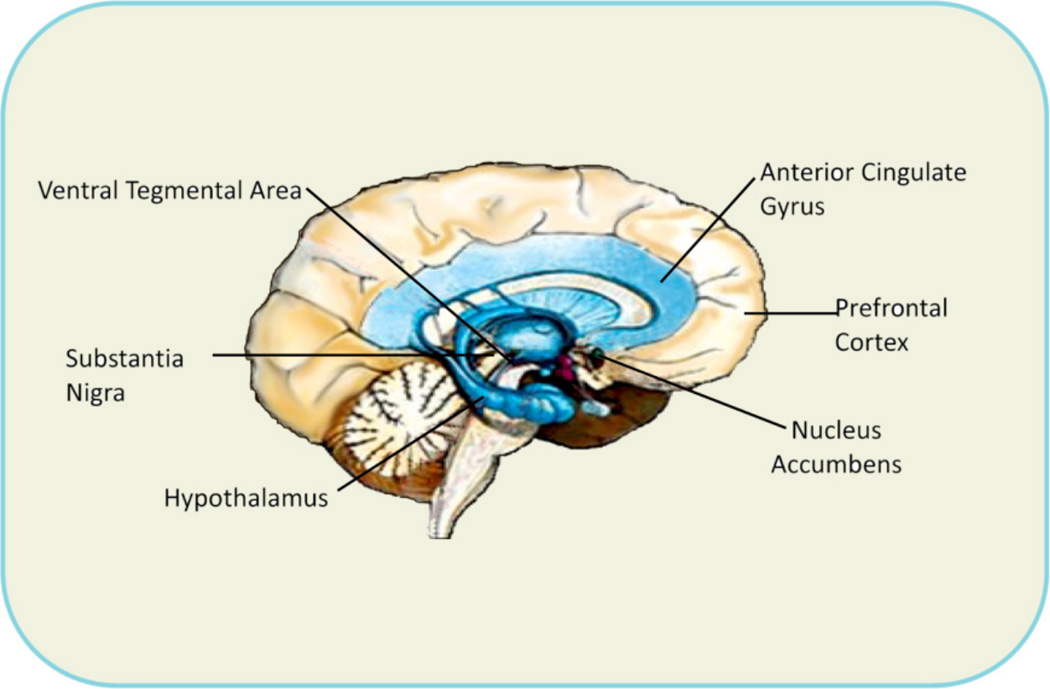

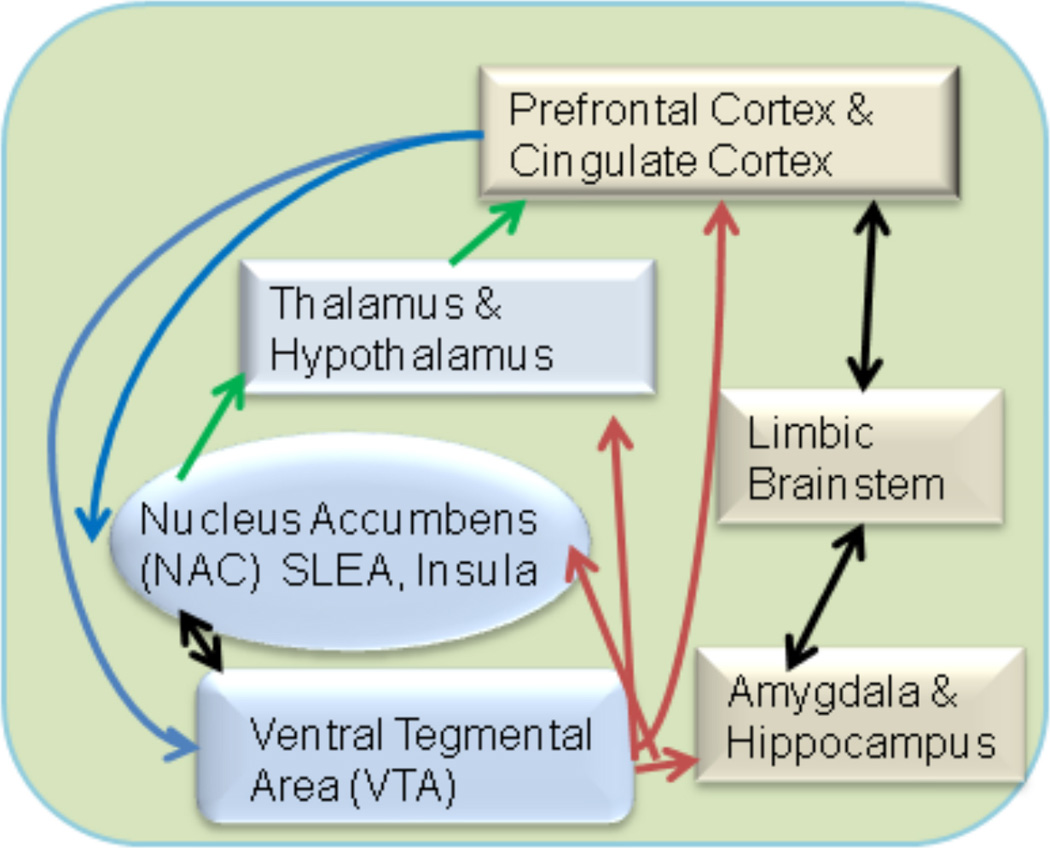
**a**: Brain Reward sites (Blum et al. [46] with permission). **b**: Extended Brain Reward Circuitry (Blum et al. [46] with permission].

**Figure 2:**
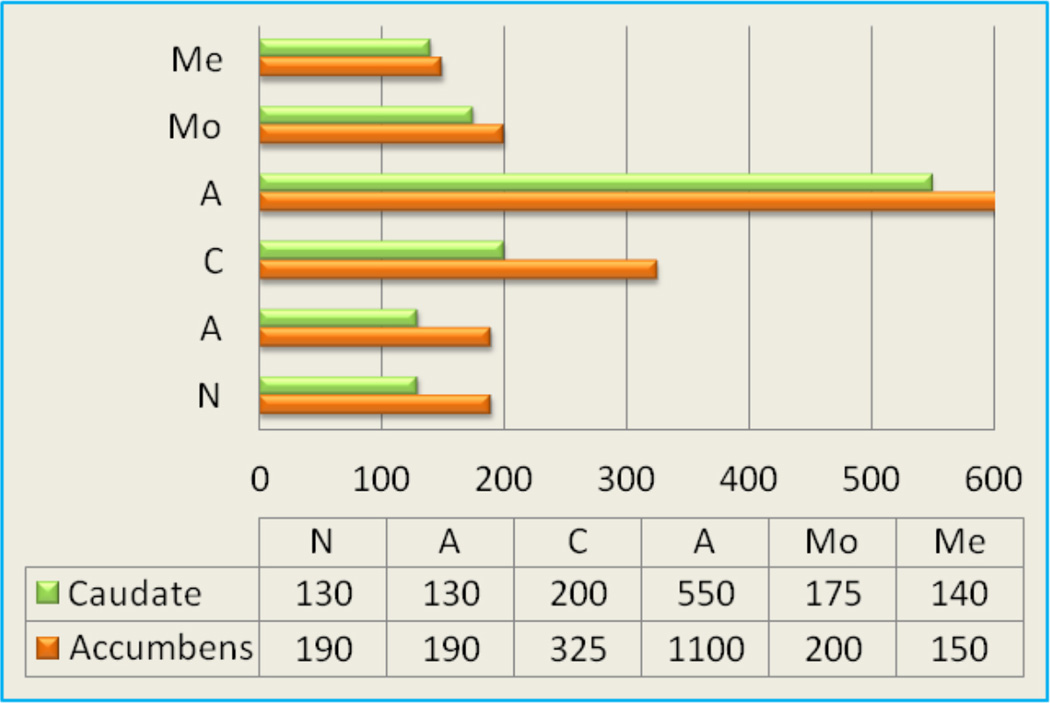
Percent increase above rest of Dopamine release in the Claudate and Accumbens as Measured by Microdialysis. Di Chiara G, Imperato A [15]-modified-Abbreviations: Me (Methadone); Mo (Morphine); A (Amphetamine); C (Cocaine); E (Ethanol); N (Nicotine) (with permission Blum et al. [46])

**Figure 3:**
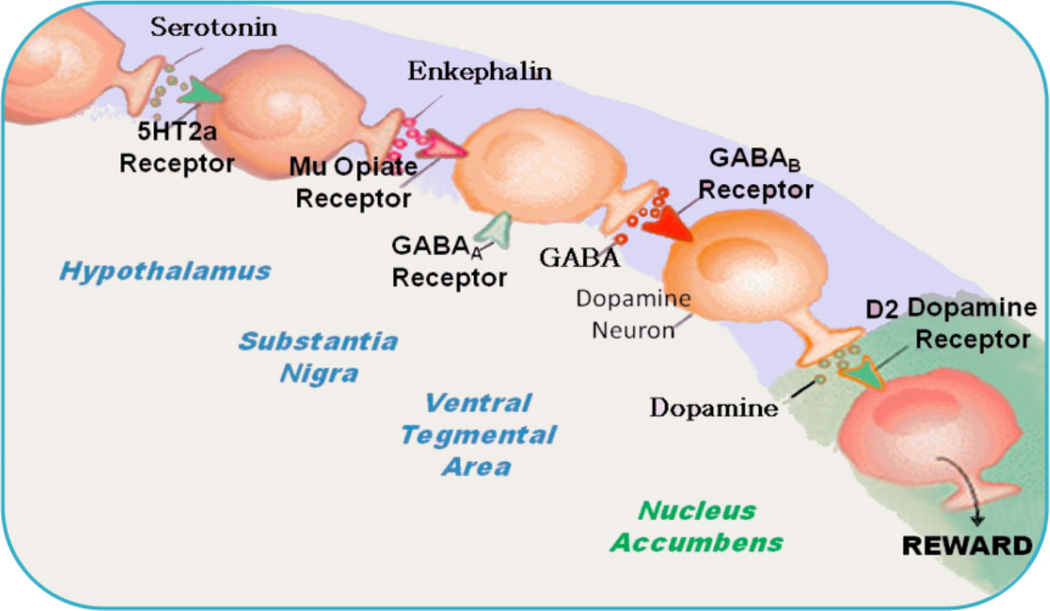
Brain Reward Cascade (with permission Blum et al. [46])

There is evidence that through the 12-step program and fellowship cross-talk between the Pre-Frontal Cortex-Cingulate (site of decision-making) and the Nucleus Accumbens (NAc) [site of craving behavior] is developed. Over half a century of dedicated and rigorous scientific research on the mesolimbic system provided insight into the neurogenetic mechanisms involved in the addictive brain and man’s quest for happiness. In brief, the site of the brain where one experiences feelings of well-being is called the Mesolimbic System and has been termed the reward center. The reward center is where chemical messages, including dopamine (DA), serotonin, enkephalins, and γ-aminobutyric acid (GABA), work together, to provide a net release of DA in the NAc. It is well known that genes control the synthesis, vesicular storage, metabolism, receptor formation, and catabolism of neurotransmitters [1–3]. Polymorphic variations in these genes can lead to an impairment of the neuronal events termed “The Brain Reward Cascade” that brings about the release of DA [4] (Figures 1a & 1b, 2, 3). A breakdown of this Cascade will lead to the dysregulation and dysfunction of DA homeostasis. Dopamine has been established as the pleasure and anti-stress molecule. Any reduction in DA function can result in a deficiency in reward that leads to substance seeking behavior [5].

After 30,000 years, *Homo sapiens* are still evolving. We are biologically predisposed to drink, eat, reproduce, and desire pleasurable experiences. Humans have evolved rapidly; a few examples of recent traits are straight black hair, blue eyes, and lactose tolerance. The switch to agrarian from hunting and gathering based societies, allowed for new advantageous mutations due to enhanced reproduction. The human genome and future generations are likely to be mosaics of the past genome, due to epigenetics. The ability to build skyscrapers and cities suggest that we are different from our closest relative *homo ergaster*. While the brain of a chimp based on cognitive tests do as well as young children, the human brain size has quadrupled over 4 million years including structures around our reward system [6].

Impairment of the mechanisms involved in reward from these natural processes lead to multiple impulsive, compulsive, and addictive behaviors governed by genetic-polymorphic antecedents [7, 8]. There is a plethora of genetic variations at the level of mesolimbic activity. Polymorphisms of these genes are candidate genes, known to predispose individuals to excessive cravings and result in aberrant behaviors. They include; serotonergic 2A receptor (5-HTT2a); serotonergic transporter (5HTTLPR); DA D2 receptor (DRD2); DA D4 receptor (DRD4); DA transporter (DAT1); the catechol-O-methyltransferase (COMT), and monoamine-oxidase (MOA) genes [9].

In 1996, the term RDS was coined to define behaviors that associated with a common genetic variant involving DRD2 polymorphisms [10, 11] as a putative predictor of impulsive, compulsive, and addictive behaviors [12–14 [see Table 1].

Having genetic polymorphisms that, for example, result in reduced serotonergic and/or dopaminergic receptor densities or an increased rate of synaptic DA catabolism, because of high catabolic genotype of the COMT gene would have reduced DA availability. Drugs of abuse, are associated with the release of DA in the mesocorticolimbic system or reward pathway of the brain [15] [Figure 2].Without adequate DA function an individual would be predisposed to self medicate with any substance or behavior that will activate DA release including alcohol, nicotine, psychostimulants, opiates, glucose, sex, gambling, and even excessive internet gaming [16].

Dopaminergic system activation induces feelings of reward and pleasure [17, 18] impacted by epigenetic factors. However, hypodopaminergic functioning can, on the other hand, trigger drug-seeking and other RDS behaviors that seem to overlap [19–21]. Gene polymorphisms can induce hypodopaminergic functioning through, for example, reduced DA receptor density, blunted response to DA, or enhanced DA catabolism in the reward pathway [22]. Cessation of chronic drug use can also induce a hypodopaminergic state that prompts drug-seeking behaviors in an attempt to address the withdrawal-induced state [23].

Acute use of psychoactive substances can induce a feeling of well-being, sustained and prolonged abuse, unfortunately, leads to a toxic “high” and result in tolerance, disease, and discomfort. Thus, excessive cravings caused by carrying the DRD2 A1 allelic genotype that causes low DA receptors are compounded by consequential drug seeking behavior. Conversely, normal DA receptor densities, do not result in craving behaviors. The goal of preventing substance abuse or excessive glucose craving then may be accomplished in genetically prone individuals by the proliferation of DA D2 receptors [24]. *In vitro*, constant stimulation of the DA receptor system with a known D2 agonist in low doses results in significant D2 receptor proliferation despite genetic antecedents [25]. In essence, in the mesolimbic system D2 receptor stimulation signals negative feedback mechanisms to induce mRNA expression and causes the proliferation of D2 receptors. In humans, based on this molecular finding natural induction of DA release could be used to generate the same D2-directed mRNA to proliferate D2 receptors in order to, attenuate craving. In fact, this worked when a form of gene therapy; DNA-directed overexpression of the DRD2 receptors induced a significant reduction in both alcohol and cocaine craving behavior in animals [26–29].

The functional RDS hypothesis of drug-seeking and drug use is that regardless of its source, the presence of a hypodopaminergic state, is a primary cause of drug seeking behavior. Genetic polymorphisms that induce hypodopaminergic functioning are the primary causal mechanism of a predisposition to chronic drug use and relapse [30]. Long-term utilization of an approach that gently activates DA might become an effective and safe treatment for RDS behaviors including; substance use disorders (SUD), attention-deficit hyperactivity disorder (ADHD), and obesity.

## Why Activate Dopamine?

Dopamine, as stated earlier, has been associated with pleasure and is the primary neurotransmitter modulating the activation of the reward system of the brain, It has been called the anti-stress molecule and the pleasure molecule [5, 31–33]. When DA is released into the synapse a number of receptors (D1-D5) are stimulated and feelings of well-being and stress reduction increase. The role of DRD2 gene in neuropsychiatric disorders and in alcoholism and other addictions [34], has been widely studied. Grasping the mechanism of motivated behavior and positive reinforcers requires an understanding of the neural circuitry of rewards [35].

A positive reinforcer is operationally defined as an event known to increase the probability of a subsequent positive response involving DA networks and drugs of abuse are considered to be stronger positive reinforcers than natural reinforcers (like food and sex) [36–38]. There is an important distinction between natural and unnatural rewards. Natural rewards include satisfaction of physiological drives (like hunger and reproduction and exploratory locomotion), and unnatural rewards are learned and involve satisfaction of acquired drives [39]. Acquired drives involve hedonic sensations and pleasure derived from alcohol, other drugs, as well as, from gambling and other risk-taking behaviors [2, 3, 36].

The reinforcing effects of drugs of abuse such as cocaine, alcohol, nicotine, food, and music are mediated in the NAc, a site within the ventral striatum. Indeed, it is believed that this structure directs motivated behaviors, elicited by natural rewards or incentive stimuli. The main tenets of positive reinforcement is that motor responses will increase in magnitude and vigor when followed by a rewarding event. Our hypothesis is that a mechanism of action for the powerful effects that drugs, music, food, and sex have on human motivation may be in part due to low DA function in the ventral striatum [40].

The human drive for the three necessary motivated behaviors, hunger, thirst, and sex, may all have common molecular-genetic antecedents that, if impaired, lead to aberrant behaviors. We hypothesize based on a plethora of scientific support that sexual activity like drugs, food, and music activates brain mesolimbic reward circuitry. Moreover, dopaminergic genes and possibly other candidate neurotransmitter-related genes and their polymorphisms affect both hedonic and anhedonic behavioral outcomes. As such we anticipate that future genetic studies of sex addiction will provide evidence for polymorphic associations with specific clustering of sexual typologies based on assessments using clinical instruments. We encourage both academic and clinical scientists to embark on neuroimaging studies of natural dopaminergic agonistic agents (like KB220Z™) to systematically target specific gene polymorphisms and normalize hyper-or hypo-sexual response [41–43].

Drug-microinjection studies have shown that opioids in brain reward regions especially in the Ventral Medial Striatum amplify the liking of sweet-taste rewards. Hedonic hot spots have been identified within the accumbens and pallidum using Fos plume mapping. These hotspots are where opioids are especially tuned to magnify the liking of food rewards. Hedonic hot spots in different brain structures may interact with each other within the larger functional circuitry that connects them [44]. Excessive hedonic liking for particular rewards might contribute to excessive consumption, and disorders such as RDS.

With this brief introduction to mesolimbic reward circuitry illustrated in Figure 3 it provides a framework for understanding the potential role of neurogenetics and neurotransmission, involving DA and the subsequent development of well-being. With this in mind we have explored the molecular neurobiology that may impact the 12 step doctrine as a model for recovery in earlier publications [45].

## Alcoholics Anonymous/Narcotics Anonymous

Alcoholics Anonymous (AA) founded in 1935 by Bill Wilson and Dr. Bob Smith (Bill W. & Dr. Bob) in Akron Ohio is an international mutual aid movement. The primary stated purpose of AA is to encourage alcoholics “to stay sober and help other alcoholics achieve sobriety”. Wilson and Smith with help from other early members developed AA’s Twelve-Step program of spiritual and character development. In 1946, The Twelve Traditions were introduced to help AA strengthen and grow. The Traditions recommend that in public media groups and members remain anonymous, include all who wish to stop drinking and altruistically help other alcoholics. The Traditions also recommend that AA members, acting on behalf of the fellowship steer clear of dogma, involvement in public issues and governing hierarchies. Subsequent fellowships such as Narcotics Anonymous have adopted and adapted the Twelve Steps and the Twelve Traditions to their respective primary purposes [47].

Although, AA generally avoids discussing the medical nature of alcoholism AA is regarded as a proponent and popularizer of the older disease theory of alcoholism [48]. The American Psychiatric Association recommended AA’s program or similar community resources, in conjunction with sustained treatment for chronic alcoholics unresponsive to brief treatment. AA’s data states that 64% drop out of AA in their first year [49, 50].

AA membership since 1935 has spread “across diverse cultures holding different beliefs and values”, including geopolitical areas resistant to grass-root movements. AA claims more than 2 million members. While there is, a difference between the 12-step program and AA/NA fellowship both can play an important part in successful recovery. In this article we have interchanged the words “fellowship” and “program” because there are those that believe they are synonymous in a real true sense. AA’s name derived from its first book, informally called “The Big Book,” originally titled “*Alcoholics Anonymous: The story of how more than one-hundred men have recovered from Alcoholism*”. While there may be a real allergy to ethanol potentially due to one’s genetic makeup, people may not be doomed. We now know the importance of environmental impact on our polymorphic genes especially those involved in the brain reward circuitry. We are also aware that many people may be able to embrace the 12-step programs but although there is no magic bullet are we getting closer to “hatching the addiction egg”? [51, 52].

## Addiction Epigenetics

An earlier simple understanding of genetics and environment held that ***P* = *G* + *E*** where P = any phenotype; G = Genes and E = environmental elements is the basis for understanding why we are not doomed because of our DNA polymorphisms. While it is believed that our genes contribute approximately 50–70% of the variance to RDS the environment seems to play a significant role in terms of gene expression and as such behaviors “normal” or aberrant”. Through extensive research during the last ten years we are beginning to understand the impact of the environment onto our genome [53].

Importantly, evidence indicates that epigenetic mechanisms are involved in drug addiction. Enzymes involved in chromatin remodeling have been recently studied. Simon-O’Brien et al. [54] found that histone deacetylase (HDAC) inhibitors (HDACi) had significant effects on ethanol intake and relapse. Specifically, they found that excessive alcohol intake of dependent (but not non-dependant) rats in the operant ethanol self-administration paradigm was significantly decreased by Sodium Butyrate (NaB) and MS-275. NaB reduced excessive drinking and prevented the escalation of ethanol intake in the intermittent access to 20% ethanol paradigm and completely blocked the increase of ethanol consumption induced by an alcohol deprivation. These results demonstrated a preventive effect of NaB on relapse.

In addition, Febo et al. [55] found that acute exposure to cocaine resulted in widespread BOLD activation in fore-and midbrain, however, chronic exposure did not. Pretreatment with the histone deacetylase inhibitor NaB restored BOLD signals in the forebrain after repeated cocaine exposure. Areas of activation included, the hippocampus/amygdala, various portions of limbic and sensory cortex and a pronounced activation in the anterior thalamus. These findings suggest that HDACi modulation after repeated stimulant exposure involves corticolimbic circuitry regulating emotion, motivation, and memory.

Since it is well-known that memory of the drug experience is an important cue for reinstatement of drug seeking and negative consequences also are cues to block reinstatement. In this regard Sen [56], reported that down-regulation of genes due to alterations in epigenetics leads to cognitive deficiencies that may play a role in the addictive process. Kenny’s group [57] suggest that there is evidence that DNA methylation plays a central role in these processes, likely by directly influencing the expression of genes involved in synaptic plasticity.

It is well-established that abuse of opiates, induce synaptic adaptation in a number of brain regions including ventral tegmental area (VTA). These adaptations may underlay the initiation and maintenance of opioid dependence and addiction in humans and animal models. Wang et al. [58], has shown that certain genes involved in glutaminergic function are altered by morphine. Through epigenetic mechanisms morphine alters a protein involved in postsynaptic density called “protein 95” (PSD-95). This protein is critically involved in the glutamatergic synaptic maturation and plasticity in the central neurons.

Scientists worldwide all agree that acute and chronic ethanol exposure may involve chromatin remodeling resulting from covalent histone modifications and DNA methylation in the neuronal circuits involving the amygdala brain region [59]. In this regard, Pandey et al. [60] revealed a novel role for amygdaloidal chromatin remodeling in the process of alcohol addiction. They further suggest that HDAC inhibitors may be potential therapeutic agents in treating alcohol withdrawal symptoms.

Importantly, microRNAs are small non-coding RNA molecules that regulate decrease or increase polypeptide formation as a function of mRNAs expression. They exert this function through base-pairing with partially complementary sequences in the 3’-UTR of target mRNAs. Since the first discovery of miR, lin-4 in *Caenorhabditis Elegans*, hundreds of miRs have been identified from humans in viruses, which have provided a pervasive layer of post-transcriptional gene regulation. The human nervous system is a rich source of miR expression, with a diversity of miR functions in fundamental neurobiological processes including neuronal development, plasticity, metabolism, and apoptosis and addiction [61]. It is also known that for example, expression of the NMDA receptor 2B (NR2B) gene is upregulated following chronic intermittent ethanol (CIE) treatment and withdrawal. This upregulation underlies behavioral alterations in addiction [62]. In some histone methyltransferases (HMTs) Qiang et al. [62] found a significant down-regulation at both the global level and the local chromatin of the NR2B gene following CIE treatment. In addition, it was also found that in the chromatin of the NR2B gene promoter, a decrease in G9a, Suv39 h1 and HDAC1-3 is responsible for the altered H3K9 modifications caused by CIE. Modifications in H3K9 show an increase in methylation with acute ethanol and a subsequent reduction of methylation during withdrawal with an increase in histone acetylation. Importantly, this is another example of how changes in H3K9 modifications in the local chromatin of the NR2B gene underlie alcohol-induced neuroadaptation. Moreover, Taqi et al. [63] revealed that following alcoholism in post-mortem tissue there was methylation in the alcoholics compared to controls. However, the methylation was found in the non-risk allele of prodynorphine-gene (PDYN). Thus, alcohol *per se* could affect even gene expression by altering the activation of PDYN transcription and vulnerability of individuals with the C, non-risk allele(s) to develop alcohol dependence.

There have been a number of animal studies showing that chronic cannabis smoking can result in molecular neurobiological modifications in the reward circuitry leading to prolonged behavioral problems. Further, this has now been confirmed by the recent work of Szutorisz et al. [64]. They found that parental exposure to the main psychoactive component of cannabis (not the same as grown cannabis) Δ(9)-tetrahydrocannabinol (THC), leads to compulsive heroin seeking behavior and changed striatal synaptic plasticity in subsequent generations. Germline THC exposure was found to decrease mRNA, with a concomitant reduction in NMDA receptor binding observed in the dorsal striatum of adult offspring. These results further suggest that THC exposure influences the molecular characteristics of the striatum and can impact offspring phenotype, leading to the augmented risk for psychiatric disorders in the subsequent generation through neuro epigenetic effects.

The effect of environmental elements on mRNA transcription is an important area of investigation. Specifically, MicroRNAs (miRNAs) are a type of non-protein-coding single-stranded RNA, typically 20–25 nt in length. Undoubtable, miRNAs play significant roles in many biological processes, including development, cell proliferation, differentiation, and apoptosis. Xu et al. [65] found that miR-212 expression level was constantly elevated during cocaine administration. Along similar lines Bahi & Dreyer [66] showed that striatal miR124a and BDNF signaling have crucial roles in alcohol consumption and ethanol conditioned reward. Zhang et al. [67] reported on the important role of miR-190 in the regulation of morphine function through its impact on OPRM1 expression.

The take-home message is that genetic variability may increase the risk of addictive behaviors in an individual and exposure to a drug results in neuroadaptations in interconnected brain circuits. In genetically susceptible individuals, these neuroadaptations underlie the transition to, and maintenance of, an addicted state. Moreover, these adaptations occur at the cellular, molecular, or epigenetic level and are associated with synaptic plasticity and modified gene expression. These effects on gene expression can occur via factors influencing translation (epigenetics) and transcription (non-coding micro RNAs) of the DNA or even RNA itself [68–70].

## Are There Epigenetic Effects in the 12 Step Programs & Fellowship?

While it may be difficult to prove because of the fact that “anonymous” prevents potential real-time exploration, there are many aspects of this important addition to the recovery process that suggest that epigenetics may have profound neurobiological influences on the reward circuitry. In the book “Molecular Neurobiology of Recovery: 12 Steps Programs and Fellowship” Blum et al. [46] adequately addresses this issue.

As we pointed out, a growing body of evidence supports the claim that the AA and the 12-step programs do work for many but not for all. One interesting notation is that those who attend regular meetings seem to adapt to recovery with “a brand new psyche”. This possible outcome will translate to a new and improved life of sobriety and or clean time and acceptance of others without judgment. We believe that through fellowship there must be powerful epigenetic effects. The “love” of another possibly even through preferential release of the bonding chemical oxytocin may induce a “***synaptic change***” leading to a degree of new found happiness. In addition, Michael Meaney and associates of McGill University, showed that effects of maternal behavior are mediated to some degree, through epigenetics. Specifically, rat mothers that display high levels of nurturing behavior, licking and grooming their pups, result in offspring that are less anxious and produce less stress hormone than pup’s raised by less caring mothers. The basic reason for this involves differential levels of methylation linked to the glucocorticoid receptor in the hippocampus. Less caring caused more methylation and reduced receptor numbers. This results in enhanced production of cortisol, with concomitant exacerbated stress [71].

### However, we must ask the following unanswered questions

1. Are there neuroplastic and lasting brain changes in regular meeting goers?
2. Is there preferential release of DA/oxytocin during AA attendance?
3. Is there proliferation of DRD2 receptors even in carriers of the DRD2 A1 (30–40% reduced D2 receptors) when these individuals regularly attend AA meetings;
4. Can we manipulate the stress level of recovering addicts with holistic approaches such as: KB220Z.to decrease methylation on the glucocorticord receptor. [KB220Z is a complex that activates functional connectivity of the brain even at rest, hyperbaric oxygenation, yoga, meditation, diet, exercise , music therapy, sound therapy, drum therapy, trauma relief therapy, cognitive behavioral therapy among other know modalities];
5. Through attendance at AA meetings can we actually proliferate D2 receptors?

D2 receptor proliferation is one example of inducing a neuroplasticity that could result in reduction of norepinephrine induced stress, reduce cravings, enhance decision-making, enhance social bonding, reduce immature defense styling (lying and or manipulation). It could also regulate Prefrontal cortices cingulate. to prevent relapse, increase focus, expand memory, augment self-esteem and confidence, reduce crime, reduce unprotected sex, enhance brain reward white/ grey matter density, and finally induce spirituality and new awakenings [45, 52].

## So Does It Make Sense to Incorporate the 12-step Program & Fellowship along with Medical Assisted Treatment (MAT)?

We should also ask the important question-If the current FDA approved drugs favor blocking DA function; Why would we want to block DA in the long-term, especially while actively seeking help through the 12 steps? The answer to this conundrum is to at least embrace the concept that blocking DA is not the best approach, until we find an appropriate form of MAT with enhanced pleasure and anti-stress to assist those in recovery. One goal would be to reduce “white knuckle sobriety” in the recovering addict.

Importantly, there is now a molecular basis for the gateway hypothesis especially for nicotine [72]. It has always been thought that young people use drugs of abuse in stages and that certain drugs like marijuana and nicotine may be gateway substance leading to heavier psychoactive drugs such as heroin and cocaine [73]. We now know that nicotine induces “hyperacetylation” in the brain and alters the expression of FOSB, which is a trigger for addiction. It is also feasible that activation of histone deacetylase (HDAC) may have some benefit in treating addiction since by doing so can decrease FOSB expression in response to cocaine. While modifying HDAC activators to target specifically the striatum would be most desirable, we must be cognizant that systemic treatment with HDAC activators or histone acetyltransferase inhibitors could be dangerous especially for cognition. In addition, e-cigarettes may be equally harmful as smoking, except for cancer potential, because it contains pure nicotine and as such could act as a gateway. These facts, have real relevance to the recovering community as it relates to continued use of tobacco and now e-cigarettes and may indeed interfere with abstinence due to setting the brain up to reinstate alcohol or other abusable licit or illicit substances through a known gateway neuro epigenetic mechanism.

## Controversy

For clarity Harvard Professor George Vaillant, surprisingly did not find evidence for effectiveness of the AA program relative to a control having no AA treatment.

### Vaillant concluded that

“*AA may be a good and comfortable fit for a few people who have a problem with alcohol, the majority of people with alcohol problems appear to do better with a different approach. We would love to see a study of why so many people dropped out of AA. We hypothesize that this may be due to the fact that AA’s theological notions of the powerlessness of humanity and of the need for a rescuing God are unpalatable not only to many atheists and agnostics but to almost all theists who are not Calvinists as well*”.

Furthermore Vaillant suggests based on his research that: *“It may also be the case that the AA philosophy of “powerlessness” over alcohol and slogans such as “one drink, one drunk”, “one is too many and a thousand is never enough” and “alcohol is cunning, baffling, and powerful” actually set people up to binge drink rather than to practice damage control when they slip up and fail to abstain as intended. More data on this topic is definitely needed*”.

### A summary of Valliant’s

- AA is a good fit for a small number of people with alcohol problems and helps them to abstain.
- AA is a poor fit for the majority of people with alcohol problems and can make some people worse.
- AA is better at creating “true believers” than it is at eliminating problem drinking.
- Whether or not AA is a good fit for a person has little if anything to do with how much a person drinks or the number of alcohol-related problems that a person has-the essential factor is personality type.
- AA is a good fit for black-and-white thinkers who accept proof by authority.
- AA is a poor fit for people who think in shades of gray and demand experimental evidence and scientific proof.

We assert it may be possible in the future to test for specific genes that would better match individuals to accepting the doctrines of AA [74]. There are examples of why AA does not work for everybody [75]. Interestingly, a PUBMED search (9-5-14) using terminology “***why alcoholics anonymous does NOT work***” did not retrieve any results. However, Kelly [75] pointed out that:

“Regarding subpopulations, current evidence suggests non-or less-religious individuals benefit as much from self-help groups as more religious individuals and women become as involved and benefit as much as men. However, participation in, and effects from, traditional self-help groups for dually diagnosed patients may be moderated by type of psychiatric comorbidity. Some youth appear to benefit, but remain largely unstudied. Dropout and nonattendance rates are high, despite clinical recommendations to attend.”

## Beyound Vaillant

Since Valliant has reviewed the potential of the 12 steps in reducing relapse, there have been many reports to the contrary revealing the importance of meditation, personality, transcendence, mindfulness and spirituality. In fact our laboratory has recently reported that as belief in spirituality rises in an individual so does remission to substance abuse [76]. There have been studies directed at understanding from a neurotransmitter level with mixed results. Whereby, Finnish scientists found no association between 5-HT-1A receptors and spiritual experiences in both patients with major depression and healthy controls [77], others did find an association. Specifically, Borg et al. [78] did find that spiritual acceptance correlated significantly with the several-fold availability of 5-HT-1A receptor density and “may explain why people vary greatly in spiritual zeal”. Along similar lines, it was found that boys and girls with the combination of presence of the short 5-HTTLPR, and homozygosity for the long AP-2beta genotype scored significantly lower on Self-Transcendence and Spiritual Acceptance [79].

Other work by Davidson’s group on mindfulness reveals the importance of mediation in terms of brain activation of the reward circuitry. They found that Expert Meditators activated to a greater degree fMRI adapted Stroop Word-Colour Task (SWCT), which requires attention and impulse control compared to novices. Understanding this could suggest that meditation coupled with enhance spiritual belief may indeed induce DA release at the VTA and cingulate gyrus that could translate to better clinical outcomes and reduced relapse [80, 81].

Certainly the genes have a role in substance use disorder as well as behavioral addictions and these subsets of RDS seem to be inheritable. However, RDS is not a monogenetic disorder with one gene causing this complex mental condition. It is polygenic with multiple inducible epigenetic effects on DNA chromatin structure and function. Belcher et al. [82] have identified three-high order personality traits that seem tied to specific brain regions and gene polymorphisms. Obviously these polymorphisms influence and effect the workings of these brain regions and ultimately reflect an individual’s personality [83] and even belief system. This could translate to either vulnerability or resilience to developing RDS.

Undoubtedly most treatment facilities would embrace the 12-step program including the helper principle [84] but its role as the only treatment option has been questioned. In spite of the fact that the use of MAT is not usually endorsed by AA/ NA or similar organizations, Chappel and Dupont [47] not only embraced the 12 step program for recovering addicts but also suggested the importance of legally prescribed treatment medications as well, especially for co-morbid psychiatric conditions. They also point out the importance of Galanters’ involvement of family members and friends in the network therapy to prevent relapse. As suggested by Scott A. Teitelbaum in his book “Addiction: A family Affair (2011) since there is no known “cure” for addiction, it is imperative that prevention must start with the family. Finally Galanter et al. [85] reported that patients more oriented toward a spiritual than a formerly religious affiliation toward other 12-step members (a spiritual awakening) associated with lower rates of substance seeking behavior.

Evidence continues to emerge regarding our understanding of brain function especially the reward circuitry and all addictive behaviors and clinicians are encouraged to review some of the select molecular neurogenetic literature [86–105].

## Summary of Molecular Neurobiology of the 12 Steps

The summary of how the molecular neurobiology effects each step has been covered in detail in Blum et al. [46]. Here we provide a brief synopsis of the main message linked to each step (see Table 2 with references).

### Step 1-We admitted that we were powerless over alcohol-that our lives had become unmanageable

While the concept of POWERLESSNESS may be controversial in the field, the first step admitting personal powerlessness over addiction is supported by the actual mechanisms involved in the neurobiological circuits of our brain. Genetic vulnerability to addiction and compulsive behaviors, compounded by epigenetically induced environmental elements. Stress and the toxic-effects of the drugs themselves induce changes in the neuroanatomy, neurophysiology, and neurochemistry of the brain that change hedonic tone, physical dependence, craving, and relapse. In essence, it is very true that indeed a person is powerless. The addicted person has no control over drug seeking and other damaging behaviors despite their denial of loss of control over drug abuse and erroneous thoughts concerning their “pseudo-power” over their unwanted behavior.

Although genetic factors play a very significant role in the process of addiction and especially in risk for developing reward dependence behaviors, as we see from experiments published above these powerful substances have strong epigenetic effects of. Those effects profoundly disrupt brain-reward homeostasis and cause an UNMANAGEABLE desire to self-administer drugs of abuse. The unmanageable desire manifests as powerlessness, an inability to control behaviors that influence every aspect of one’s life.

### Step 2-Came to believe that a Power greater than ourselves could restore us to sanity

Sanity (sound judgment) or insanity (repetitive behavior despite the harm) may be impaired even at birth and could be due to deficient brain reward circuitry function especially resulting in a hypodopaminergic trait. This poor judgment could be a cause of aberrant substance seeking behavior in the face of harm’s way. Poor decisions compounded by environmental factors including drug availability, non-nurturing parents, social-economic burdens, and stress. Importantly the ability to behave sanely also may be impacted by an individual’s relationship with a power greater than themselves. In terms of relapse, it is well known that the prefrontal cortex and cingulate gyrus are critical areas of the brain involved in relapse regulation. Poor judgment caused by impairments in the neurochemical functioning of these regions due to genes and/ or toxic substances and/or behaviors impede recovery and induce relapse. Understanding the molecular biology of the brain reward system (genes and environment) highlights the importance of positive input from fellowship (self-help) programs and other treatment modalities. Positive input from fellowship can offset unwanted gene expression, lift spirits, and assist in enabling the individual to achieve a state of sanity and make right choices.

### Step 3-Made a decision to turn our will and our lives over to the care of God as we understood him

Will-power is difficult to control, especially in individuals born with a compromised reward system, and low levels of endorphins. Genetically predisposed individuals seek out drugs such as alcohol, heroin, cocaine, nicotine, and even sugar. These substances all activate the reward substrates (like serotonin, enkephalins, GABA and DA pathways) and provide a pseudo temporary feeling of well-being (so called “normalization”). Willpower is based on both the interplay of genes and environmental elements in society. Stress as an adult and surprising during the prenatal phase are environmental elements. This early stress can lead to aberrant substance use disorders in adult life as seen with epigenetic effects on Glucocorticoid receptor express. Since it is difficult to fight the hard wiring of our brain reward circuitry, for the recovering addict it seems obvious to look for reward outside of our genome (i.e. alcohol, drugs, sex, and food).

### Step 4-Made a searching and fearless moral inventory of ourselves

Fearless moral inventory must include the drug of choice and other RDS related behaviors because the phenotype is not any particular drug or behavior; it is indeed RDS. However, the inventory the individual is completing cannot be “right” or “wrong,” because it his/her *own* evaluation of self and list of resentments. Moreover, the *Big Book* states (How It Works, Page 60), “No one among us has been able to maintain perfect adherence to any of these principles. The point is that we are willing to grow along spiritual lines. The principles we have set down are guides to progress. We claim spiritual progress rather than spiritual perfection.” Several “fourth steps” may be taken by an individual over the course of his/her sobriety. Moreover, it is almost impossible for addicts in early recovery, to embrace Step 4. Impairments of brain reward circuitry are protracted and amplified during withdrawal and early recovery, for example, in alcoholics, heroin addicts, and cocaine addicts. Unfortunately, this could be due to the chronic abuse of these powerful substances as epigenetic phenomena, as well as, possible inherited reward gene polymorphisms that occur at birth. It has been reasoned that one therapeutic target involves continued natural DA D2 activation as reflected in the preliminary fMRI research being conducted in China using KB220Z [106].

### Step 5-Admitted to God, to ourselves, and to another human being the exact nature of our wrongs

This step involves the consideration of our issues with “getting high”, as well as, the toxic effects of continual exposure to these powerful substances. Their impact on brain reward networks is indeed physiological (e.g., increase in brain DBI). Physiological changes can result in psychological effects (anxiety and aggression) that are behind behaviors with harmful and sometimes fatal consequences not only to ones-self but others.

### Step 6-Were entirely ready to have God remove all these defects of character

Although it is possible to define character in a moralistic sense, it is very difficult to assign responsibility for defects of character and the wrong-decisions and consequence since character is shaped by genetic (evolutionary) forces far beyond a person’s control. With this stated it is argued that environmental elements especially in childhood may also require rethinking in terms of blame and or even praise of an individual act. This idea supports the idea in the sixth step that the removal of character defects is the province of a higher power. Clinicians should be cognizant that for the individual, achievement of this step requires deep character analysis, painful realization, and ability to dissociate oneself (present) from the past self. It should also be noted that carriers of the DRD2 gene polymorphism (risk for addiction) will have great difficulty being honest.

### Step 7-Humbly asked Him to remove our shortcomings

Being humble must be accompanied with both gratitude and grace. The concept of ‘turning it over’ and let GOD remove our shortcomings is not easily accomplished. To be humble is akin to having gratitude for the things we have the idea of moving forward. Statements of spiritual faith and being humble challenge the recovering person to face the fact that good intentions and honest effort alone will not always succeed in getting him or her what is truly wanted from life. In turn, and supported by genetic predisposition, this could lead to chronic depression and relapse. However, the 12-step program and the traditions together ask the person to believe that evil and brutishness, injustice and cruelty will not necessarily win out in the end. Being humble and having faith, advocates neither passivity nor hopelessness; on the contrary, they express the belief that our shortcomings can be removed by our willingness to believe that things can work out for the best in the long term. Having positive feelings about GOD translates to positive epigenetics that enhance the chances that we could remove our shortcomings by expressing “good” genes rather than “bad” genes.

### Step 8-Made a list of all persons we had harmed, and became willing to make amends to them all

It is not easy to make amends especially to people who are not only our friends but people whom we love. Step 8 does not come early in one’s sobriety but only after periods of being clean and sober. However, once an individual accomplishes this arduous task he or she will be able to move forward on the path to recovery. In terms of connecting the dots, it is important for clinicians to realize the old adage of “Birds of a feather flock together” may be an effect of a genetic association. By virtue of friends seeking friends who, not only have similar characteristic (maybe even drinking, drugging, and eating), but similar genotypes, such as the DRD2 A1 allele So that when the alcoholic, for example, is asked to make amends and also eliminate certain friends that would not be conducive to their recovery, we need to be cognizant of going against the genetic grain. Thus, on a molecular neurobiological level, it is easily said but not easily done. A form of happiness is that people live in social networks that are comfortable. Making amends for the hurt may not reestablish trust but may help assuage guilt and shame. Here it may be helpful to consider the genetic predisposition of families to RDS behaviors.

### Step 9-Made direct amends to such people wherever possible, except when to do so would injure them or others

It is not easy to achieve happiness and peace especially when the alcoholic or addict is faced with taking responsibility for hurting others with whom he or she had a relationships while drinking and drugging. An obvious source of injury to relationships caused by addiction is the “abandonment” of a spouse or significant other for alcohol and/or drugs. Victims of RDS must take responsibility for this abandonment of loved ones. Furthermore, addicts may have been very abusive (both physically and emotionally) during their active addiction. In Step 8 before any amends can be made, the addict is asked to take an inventory of all persons harmed, which can easily evoke intense feelings of guilt and shame. It also requires overcoming denial and being willing to make amends. In Step 9, the achievement of making amends (except where doing so would cause no further injury) is subject to correlations among genes, friendships, and relationships. As noted in the research summarized above, relationships and happiness are based on neuronal hard wiring, and this presents both a formidable challenge and clarity as to how to achieve healing during recovery. The degree to which the person can make amends to others (without harm or hurt) is tantamount to a healthy recovery, and importantly, the attainment of happiness. Making amends can be facilitated by the active natural release of DA in reward centers of the brain.

### Step 10-Take personal inventory and admit to being wrong

The tenth step can be a pressure-relief valve. Addicts work this step while the day’s ups and downs are still fresh in mind. They list what they have done and try not to rationalize their actions. The first thing they must do is stop! Then they must take the time to allow themselves the privilege of thinking. They work this step continuously. It presents a way of avoiding grief. The individual monitors feelings, emotions, fantasies, and actions. By constantly looking at these things they may be able to avoid repeating the actions that make them feel bad (*Narcotics Anonymous Basic Text*, Chapter 4/Step 10). Step 10 is the maintenance for Steps 4 and 5 and “encourages the taking of a personal inventory, which, for recovering persons, should be a daily process”. It is important that addicts realize that if they do carry a genetic risk, for example, the DRD2 A1 allele with 30–40% less D2 receptor density, taking inventory and feeling good about it is a temporarily “dopamine fix”. As such, addicts must continue to “work the steps” on a daily basis to replenish DA.

### Step 11-Sought through prayer and meditation to improve our conscious contact with God, *as we understood Him*, praying only for knowledge of His will for us and the power to carry that out

Doing the work required in Step 11 continuously through both the meditative and prayer process increases the release of DA at the synaptic level. In addition, working Step 11 on a daily basis will offset the genetically induced “hypodopaminergic brain function” by continued DA release in the synapse. Increased DA will result in a subsequent proliferation of DA D2 receptors even in carriers of the DRD2 A1 allele and other reward gene polymorphisms. The increase in D2 receptors translates to enhanced DA function, which will ultimately promote greater confidence in the recovering addict, enabling a better understanding of the written word of the twelve-step fellowship. This will lead to an anti-stress effect and as such reduce the chance for relapse especially in dysfunctional and co-dependent families.

### Step 12-Having had a spiritual awakening as the result of these steps, we tried to carry this message to alcoholics and to practice these principles in all our affairs

Step 12 occurs when the recovering person had done the work, and truly understands all the preceding steps in the program. It has been said that working all the steps will allow an individual to have spiritual awakening. We point out that for people who have addiction is dependent on both genes and environmental conditions, attaining this awakening may be more or less difficult. One of the most fulfilling experiences one could get is sharing emotions with others especially as it relates to carrying the message of the fellowship to other addicts. It is important to realize this experience may be impacted by the synthesis and release of the brain chemical oxytocin. Unfortunately, independent of one’s genetic makeup, alcohol and opiates significantly impair the synthesis and release of this important human bonding neuropeptide. Finally, clinicians should be cognizant that any lifestyle change is significantly impacted by both polymorphic genes and traumatic events.

**Figure 5:**
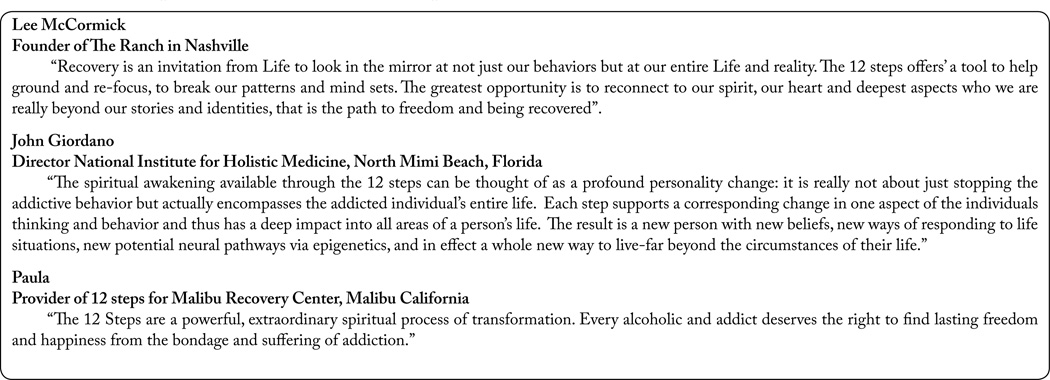
Professional’s views of the 12 Step Program & Fellowship.

Although still controversial one way to sum up the benefits of the 12 step program & fellowship is embedded and so reflected in these statements by addiction professionals working in the field of addiction medicine (see Figure 5).

## Conclusion

While it would be easy to say that all addicted individuals would benefit from the 12 step doctrines, this may not be the case. In fact when it comes to spirituality there are a number of genes and associated polymorphisms that load onto one’s beliefs related to GOD [46, 74, 79, 137, 287–289]. One member stated that “The program is perfect and it does not fail-people will.” (*Anonymous*) .

As we stated before “*Finding happiness may not only reside in our genome but may indeed be impacted by positive meditative practices, positive psychology, spiritual acceptance, love of others and self, and taking inventory of ourselves-one day at a time*” [46].

## Figures and Tables

**Table 1:** Reward Deficiency Syndrome (from Blum et al. [45] with permission).

Addictive Behaviors		Impulsive Behaviors		Obsessive Compulsive Behaviors	Personality Disorders
Substance Related	Non Substance Related	Spectrum Disorders	Disruptive Impulsive		
Alcohol	Thrill seeking (novelty)	Attention-deficit Hyperactivity	Anti-social	Body Dysmorphic	Paranoid
Cannabis	Sexual Sadism	Tourette and Tic Syndrome	Conduct	Hoarding	Schizoid
Opioids	Sexual Masochism	AuThism	Intermittent Explosive	Trichotillo-mania (hair pulling)	Borderline
Sedatives/Hypnotics	Hypersexual		Oppositional Defiant	Excoriation (skin picking)	Schizotypal
Stimulants	Gambling		Exhibitionistic	Non-suicidal Self-Injury	Histrionic
Tobacco	Internet Gaming				Narcissistic
Glucose					Avoidant
Food					Dependent

**Table 2:** The Molecular Neurobiological Summary of the 12 Steps (taken with permission from Blum et al. [46]).

STEP	Summary with References
First-Powerless	While the concept of POWERLESSNESS may be controversial in the field, the first step admitting personal powerlessness over addiction is supported by the actual mechanisms involved in the neurobiological circuits of our brain. It begins with genetic vulnerability to addiction and compounded by epigenetically induced environmental elements. Stress and the toxic effects of the drugs and compulsive behaviors themselves induce changes in the neuroanatomy, neurophysiology, and neurochemistry of the brain that effect hedonic tone, physical dependence, craving and relapse. Although genetic factors play a very significant role in the process of addiction and especially in risk for developing reward dependence behaviors, there are strong epigenetic effects of powerful substances. Those substances like alcohol etc. profoundly affect brain reward homeostasis and an unmanageable desire to self-administer drugs of abuse. This leads to powerlessness, an inability to control behaviors in face of harm that ultimately influences every aspect of one’s life [4, 104, 107–135].
Second-Restore Us to Sanity	Sanity (sound judgment) or insanity (repetitive behavior in spite of harm) may be impaired even at birth and could be due to deficient brain reward circuitry function especially resulting in a hypodopaminergic trait. This poor judgment could be a root cause for aberrant substance-seeking behavior in the face of harm’s way. This becomes further complicated when other environmental factors are present including drug availability, non-nurturing parents, social economic burdens and stress. Importantly the ability to behave sanely also may be impacted by an individual’s relationship with a power greater than themselves. In terms of relapse, it is well known that the prefrontal cortex and cingulate gyrus are very important brain regions that could regulate relapse. Poor judgment stemming from impairments in the neurochemical functioning of these regions due to genes and/or toxic substances and/or behaviors, impedes recovery and induces relapse. Understanding the molecular biology of the brain reward system (genes and environment) highlights the importance of positive input from fellowship (self-help) programs and other treatment modalities that can offset unwanted gene expression, lift spirits, and assist in enabling the individual to achieve a state of sanity and make good choices [5, 9, 16, 74, 78, 79, 104, 136–179].
Third-Turn our lives over to GOD	Will-power is not simple to control, especially if you are born with a compromised reward system, especially low levels of endorphins. Genetically predisposed individuals seek out drugs such as alcohol, heroin, cocaine, nicotine, and even sugar, because these substances all activate reward substrates (i.e., enkephalins, DA pathways) and provide a pseudo temporary feeling of well-being (so called “normalization”). Will power is based on both the interplay of genes and environmental elements in society. This includes stress as an adult and surprising during the prenatal phase. This early stress could lead to aberrant substance use disorders in adult life. Since it is not easy to fight the hard wiring of our brain reward circuitry, for the recovering addict it seems obvious to look for reward outside of our genome [i.e. alcohol, drugs, sex, and food] [180–186].
Fourth-Fearless & Moral Inventory of Ourselves	Fearless moral inventory must include not only the drug of choice but other Reward Deficiency Syndrome related behaviors. This is so because the phenotype is not any particular drug or behavior of choice; it is indeed Reward Deficiency Syndrome. However, the inventory the individual is completing cannot be “right” or “wrong,” because it his/her *own* list of resentments, and evaluation of self. Moreover, the *Big Book* states, “No one among us has been able to maintain perfect adherence to any of these principles. The point is that we are willing to grow along spiritual lines. The principles we have set down are guides to progress. We claim spiritual progress rather than spiritual perfection.” Several fourth steps may be taken by an individual over the course of his/her sobriety. Moreover, it is literally almost impossible for early recovering addicts to embrace Step 4 due to protracted abstinent impairments of brain reward circuitry, for example, in alcoholics, heroin addicts, and cocaine addicts. Unfortunately this could be due to the chronic abuse of these powerful substances as an epigenetic phenomena, as well as possible inherited reward gene polymorphisms that occur at birth. It has been reasoned that one therapeutic target involves continued natural DA D2 activation as reflected in the preliminary fMRI research being conducted in China using KB220Z [51, 15, 24, 29, 31, 41, 67, 102, 106, 115, 187–195].
Fifth-Admitted Exact Nature of our Wrong Doings	Understanding our natural desire to obtain pleasure states and to admit “wrong doing” to God, ourselves, and those around us is no simple task and involves the consideration of not just our issue with “getting high” but rather with the toxic effects produced in the brain by continual exposure to these powerful substances. Their impact on brain reward networks is indeed physiological (e.g., increase in brain DBI). This can result in mental effects (anxiety and aggression) that also result in harmful behaviors with harmful and sometimes fatal consequences not only to one-self but to others [187, 196–208].
Sixth-Removing Defects in Character	Although it is possible to define character in a moralistic sense, it is very difficult to assign responsibility for defects of character and the bad decisions and consequence since character is shaped by genetic (evolutionary) forces far beyond a person’s control. With this stated it is argued that environmental elements especially in childhood may also require rethinking in terms of blame and or even praise of an individual act. This idea supports the idea in the sixth step that the removal of character defects is the province of a higher power. Clinicians should be cognizant that for the individual, achievement of this step requires deep character analysis, painful realization, and ability to dissociate oneself (present) from the past self. It should also be noted that carriers of the DRD2 gene polymorphism (risk for addiction) will have great difficulty in achieving honesty [209–225].
Seventh-Removing Shortcomings	Being humble must be accompanied with both gratitude and grace. The concept of ‘turning it over’ and let GOD remove our shortcomings is not easily accomplished. To be humble is akin to having gratitude for the things we have the idea of moving forward. Statements of spiritual faith and being humble challenge the recovering person to face the fact that good intentions and honest effort alone will not always succeed in getting him or her what is truly wanted from life. I n turn and supported by genetic predisposition, this could lead to chronic depression and relapse. However, the 12-step program and the traditions together ask the person to believe that evil and brutishness, injustice and cruelty will not necessarily win out in the end. Being humble and having faith advocates neither passivity nor hopelessness; on the contrary, they express the belief that our shortcomings can be removed by our willingness to believe that things can work out for the best in the long term. Having positive feelings about GOD translates to positive epigenetics which enhance the chances that we could remove our shortcomings by expressing “good” genes rather than “bad genes” [10, 11, 99, 226–244].
Eighth-Making Amends of Harms	It is not easy to make amends especially to people who are not only our friends but people whom we love. Step 8 does not come early in one’s sobriety but only after periods of being clean and sober. However, once an individual accomplishes this arduous task he or she will be able to move forward in the path of recovery. In terms of connecting the dots, it is important for clinicians to realize that the old adage of “Birds of a feather flock together” may be inheritable by virtue of friends seeking friends who not only have similar characteristic (maybe even drinking, drugging, and eating), but similar genotypes, such as the DRD2 A1 allele. So that when the alcoholic, for example, is asked to make amends and also eliminate certain friends that would not be conducive to their recovery, we need to be cognizant about going against the genetic grain. Thus, on a molecular neurobiological level, it is easily said but not easily done. A form of happiness is that people live in social networks that are comfortable. Making amends for the hurt may not reestablish trust but may help assuage guilt and shame. Here it may be helpful to consider the genetic predisposition of families to Reward Deficiency Syndrome behaviors [46, 104, 165, 216, 245–267].
Ninth-Direct Amends to Such People without Injury to Them	It is not easy to achieve happiness and peace especially when the alcoholic or addict is faced with taking responsibility for hurting others with whom he or she has relationships while drinking and drugging. An obvious source of injury to relationships caused by addiction is the “abandonment” of a spouse or significant other for alcohol and/or drugs. Victims of Reward Deficiency Syndrome must take responsibility for this abandonment of loved ones. Furthermore, addicts may have been very abusive (both physically and emotionally) during their active addiction. Before any amends can be made, the addict is asked in Step 8 to take an inventory of all persons harmed, which can easily evoke intense feelings of guilt and shame. It also requires overcoming denial and being willing to make amends. In Step 9, the achievement of making amends (except where doing so would cause no further injury) is subject to correlations among genes, friendships, and relationships. As noted in the research summarized above, relationships and happiness are based on neuronal hard wiring, and this presents both a formidable challenge and clarity as to how to achieve effective healing in recovery. The degree to which the person can make amends to others (without harm or hurt) is tantamount to a healthy recovery, and importantly, the attainment of happiness. This can be facilitated through the positive natural release of DA in reward centers of the brain [34, 137, 268–271].
Tenth-Take Personal Inventory and Admit to being wrong	The tenth step can be a pressure-relief valve. Addicts work this step while the day’s ups and downs are still fresh in mind. They list what they have done and try not to rationalize their actions. The first thing they must do is stop! Then they must take the time to allow themselves the privilege of thinking. They work this step continuously. It presents a way of avoiding grief. The individual monitors feelings, emotions, fantasies, and actions. By constantly looking at these things they may be able to avoid repeating the actions that make them feel bad (*Narcotics Anonymous Basic Text*, Chapter 4/Step 10). Step 10 is the maintenance step for Steps 4 and 5 and encourages the taking of a personal inventory, which, for recovering persons, should be a daily process. It is important that addicts realize that if they do carry a genetic risk, for example the DRD2 A1 allele among other gene deficits with 30–40 % less D2 receptor density, taking inventory and feeling good about it is a temporarily “dopamine fix.” As such, addicts must continue to “work the steps” on a day-to-day basis to replenish DA. [25, 272–274].
Eleventh-Prayer & Meditation to Contact God	Doing the work required in Step 11 continuously through both the meditative and prayer process increases the release of DA at the synaptic level. In addition, working Step 11 on a daily basis will offset the genetically induced “hypodopaminergic brain function” by continued DA release in the synapse. Increased DA will result in a subsequent proliferation of DA D2 receptors even in carriers of the DRD2 A1 allele and other reward gene polymorphisms. The increase in D2 receptors translates to enhanced DA function, which will ultimately promote greater confidence in the recovering addict, enabling a better understanding of the written word of the twelve-step fellowship. This will lead to an anti-stress effect and as such reduce the chance for relapse especially in dysfunctional and co-dependent families [25, 32, 42, 156, 169, 258, 272, 274, 275–278].
Twelfth-Spiritual Awakening & Carrying the Message to Others	**Step 12**-occurs when the recovering person had done the work, and truly understands all the preceding steps in the program. It has been said that working all the steps will allow an individual to have spiritual awakening. We point out that for people who have addiction is dependent on both genes and environmental conditions, attaining this awakening may be more or less difficult. One of the most fulfilling experiences one could get is sharing emotions with others especially as it relates to carrying the message of the fellowship to other addicts. It is important to realize this experience maybe impacted by the synthesis and release of the brain chemical oxytocin. Unfortunately, independent of one’s genetic makeup, alcohol and opiates significantly impair the synthesis and release of this important human bonding neuropeptide. Finally, clinicians should be cognizant that any lifestyle change is significantly impacted by both polymorphic genes and traumatic events [79, 279–286].
